# Editorial: Children Listen: Psychological and Linguistic Aspects of Listening Difficulties During Development

**DOI:** 10.3389/fpsyg.2020.584034

**Published:** 2020-10-28

**Authors:** Birgitta Sahlén, K. Jonas Brännström, Viveka Lyberg Åhlander, Mary Rudner

**Affiliations:** ^1^Logopedics, Phoniatrics and Audiology, Department of Clinical Science, Lund University, Lund, Sweden; ^2^Department of Behavioural Sciences and Learning and Linneaus' Center HEAD, Linköping University, Östergötland, Sweden

**Keywords:** listening skills, listening effort, speech perception, noise, children's cognition

The goal for this Research Topic was to advance the scientific state of the art by collecting empirical and theoretical contributions relating to listening in children. Empirical articles that apply methods including behavioral, psychophysical, and neuroimaging approaches to the study of any aspect of listening in children were welcomed. The plethora of the articles included in the present topic illustrate the complexity and the broad areas of research necessary to understand listening in children. The many avenues of research in the field suggest the need for continuous development to a coherent theoretical model that can be used to test predictions about listening and listening effort in children. In the following, we briefly summarize the 24 contributions.

## Effects of Noise and Children's Own Perceptions of the Learning Environment

Listening in context, i.e., the intentional act of focusing attention on a particular source of auditory information in a specific multimodal setting is crucial for linguistic, cognitive, and social development. At the same time, listening often takes place to the accompaniment of background noise, and even low levels of background noise have been found to reduce listening comprehension in children. The preschool learning environment is considered to be particularly noisy. Few studies have, however, reported on preschoolers' own perceptions of their learning environment. McAllister et al. in a comprehensive interview study, explore how preschool children in Finland and Sweden, describe the preschool environment in relation to noise, voice, and verbal communication. Results were similar across countries; preschool children are well aware of high noise levels, they blame other children for making noise and shouting and for impaired communication and effects on hearing. Interestingly, they seem less aware of effects of noise on their own voice. Astolfi et al. reported on the relationship between acoustical measurement of classrooms and first graders' perceived well-being and noise disturbance. Children are less happy with themselves and have less fun with increasing levels of noise; feelings and perceptions that may have a serious impact on motivation and learning in the classroom. Prodi et al. investigated the effects of different types of noise, age, and gender on 11–13 year old children's speech intelligibility and sentence comprehension. Classroom noise was found to have the worst effect on both tasks. A developmental effect was seen, which depended on the task and listening condition in both tasks. Girls were more accurate and quicker to respond in most listening conditions. It is evident that dynamic models are needed to capture the complex interaction of task demands and individuals' capacity, perceived effort and motivation in the class-room. Listening in background noise uses cognitive resources and has an inevitable effect on listening effort. Theoretical frameworks of listening effort suggest that reverberation may have similar impact on listening effort and fatigue as noise. Interestingly, findings by Picou et al. suggest that increased moderate reverberation has no effect on listening effort or fatigue. This finding together with findings showing that the association between behavioral measurements of listening effort and participants' own ratings of perceived listening effort are weak emphasize the need for further testing of theoretical assumptions.

## The Role of Perceptual, Linguistic, and Cognitive Skills for Listening and Academic Success in Children With Hearing Loss (HL) and Auditory Processing Disorder—Indications for Intervention and Therapy

Children with poor perceptual, linguistic and cognitive skills and who are without the correct support are at an even greater risk of listening difficulties than those with typical development (TD). Bilingual children with weak school language (L2) are often considered particularly vulnerable to background noise. We should, however, be careful explaining vulnerability to noise by bilingualism.  Andersson et al. highlight the need to look beyond bilingualism and to consider explanations to academic struggle. In their comprehensive study of Swedish school children bilingualism alone predicted 38% of the variance in language scores. With information added on parental education, school characteristics, and enrolment in the school's recreation center the unique contribution of bilingualism was reduced to 9%. In the classroom setting, a vulnerable group of children comprises those with auditory processing disorder (APD). These children appear to have normal hearing sensitivity but still have listening difficulties. There is, however, a high co-existence of APD with other disorders affecting language, reading and attention, and large variation in the presentation of the difficulties. It is therefore essential to identify subgroups to inform clinical intervention for the individual child. Sharma et al. identified four different clusters of children with suspected APD. Differences in working memory capacity, phonological processing, and non-verbal intelligence were the main skills that characterized these clusters. The need for assessing a large range of skills in these children is thus evident according to the authors. Further examples of groups of children that encounter specific challenges in noisy environments are children with hearing loss (HL), with cochlear implants (CI) and/or hearing aids, children with developmental language disorder (DLD). Children in these groups can have excellent speech recognition in quiet, but still experience unique challenges when listening to speech in noisy environments. Von Koss-Torkildsen et al. investigated how speech-in-noise (SiN) perception relates to individual differences in cognitive and linguistic abilities in children with HL and typically developing (TD) children with the Hearing in Noise Test (HINT). For the full sample, language ability explained a significant amount of variance in HINT performance beyond speech perception in quiet and, that language ability was a significant predictor of HINT performance for children with CI, Hearing aids, and DLD, but not for children with TD. The authors, as most other authors in this topic, conclude that technologies that support audibility together with language-specific early interventions to help improve children's capabilities to handle noisy classroom environments are crucial for outcome. Several other contributions address children with hearing loss. For example, Socher et al. explored why children with HL often perform more poorly compared to their hearing peers, on tests of socio-pragmatic skills. In their study, significant differences between participants with HL and children with TD were found on a measure connected to theory of mind. Further, a measure of verbal fluency was correlated with three sub-measures of pragmatic language ability. Thus, children with a better developed semantic network may be able to use language in a more flexible way for communication, which is of great importance when the source signal is degraded as for children with HL. Lexical intervention may thus promote vocabulary growth and comprehension to support interaction and learning in children with HL. This is also emphasized by Wass et al. who found that receptive vocabulary was the most influential predictor of reading comprehension in 29 11–12-year-old Swedish children with profound HL using CI. Education should thus, focus both on broadening and deepening of the children's vocabularies and comprehension of spoken language. That optimal classroom acoustics help children perceive also the minute details of language and thus promote understanding is argued by

Kirkhorn Rødvik et al., who explored perception and production of speech in children with CI compared to TD children. They found that for the participants with CIs, consonants were mostly confused with consonants with the same voicing and manner and that voiced consonants were more difficult to perceive than unvoiced consonants. As is commonly reported, vowels were perceived more easily compared to consonants. Authors conclude that classroom acoustics with high reverberation times can easily hamper language comprehension due to masking effects.

Deroche et al. examined the production and perception of lexical tones (F0) in Mandarin speaking children with cochlear implants (CI). They found that children with CI relied more on durational cues than F0-contours to produce and perceive lexical tones than their peers with normal hearing. This indicates a link between production and perception also in children with CI who have poorer access to auditory feedback during production.

Further, predictions of language development are studied, for example by Ching et al., who investigated to what extent cognitive ability at 5 years of age predicted language development from 5 to 9 years of age in a population-based sample of children with HL who participated in the Australian Longitudinal Outcomes of Children with Hearing Impairment (LOCHI) study. Digit span score at 5 years was a significant predictor of receptive and expressive language at 9 years, even when non-verbal IQ and 5-year-old receptive vocabulary were accounted for. The authors argue that these findings shed light on the unique role of early verbal working memory in predicting the development of language and vocabulary skills in children who use hearing aids. Further, Jing et al. investigated the association of rhyme awareness, a common index of phonological awareness, with vocabulary and working memory in a small group of North American children (*n* = 6) with CI. While associations were statistically significant in a larger group of children with TD (*n* = 15), only the association between rhyme awareness and working memory was significant in the children with CI. As for the production of emotional tone, Chatterjee et al. conclude that access to acoustic hearing in early childhood is important and speech prosody should be included in speech therapy. The authors compared acoustic characteristics of happy and sad vocal emotions produced by North American prelingually deaf school-aged children with CI during sentence reading with those produced by peers with TD and adults with normal hearing and postlingually deaf adults with CI. They found that all four groups differ in voice pitch between the two emotions produced, but that the difference was smallest for the children with CI.

Etiology of the hearing loss may also play a role for the development of language and cognition in children with HL. Löfkvist et al. studied the role of congenital cytomegalovirus (cCMV) infection on executive function. cCMIV is the most common cause of progressive HL and associated with behavioral anomalies. Authors did not find any significant difference in executive function between two small groups of Swedish child CI users, one with cCMV and one with genetic HL. However, they did find that pragmatic skills were reduced in the cCMV group and suggest that this may hamper academic success. Word reading and spelling in children with HL are also addressed since listening skills are not only essential for spoken language development but also for the development of reading and writing. Phonological processing skills have been considered predictive of good word decoding. In the paper by Gokula et al. the general co-existence of perceptual, cognitive, and linguistic deficits in children with word reading difficulties is highlighted. A comprehensive test battery designed to assess their auditory processing, visual attention, digit memory, phonological processing, and receptive language is used. Six percent of children with word reading difficulties have deficits across all measured tasks. The results thus emphasize the significant individual variability inherent in children with word reading difficulties and the importance of thorough and comprehensive assessments of reading skills. As for writing skills, the findings by Gärdenfors et al. conclude that spelling strategies in children with HL mostly rely on auditory input but the children with CI apply visual strategies when necessary.

## Maturation of Speech Perception, Psychophysical, and Neuroimaging Approaches to Listening

A range of interesting studies in this topic address maturation of masked and unmasked speech perception (i.e., Leibold and Buss, McCreery et al., Walker et al., and MacCutcheon et al.) and its relation to linguistic and cognitive development in children with TD and HL.

In their review article, Leibold and Buss summarize evidence showing that the ability to recognize masked speech develops over an extended period during maturation. Generally speaking, children have greater difficulty than adults. In steady-state noise, this difference persists until the age of about 9–10 years but when the masker is speech the difference extends into adolescence. The authors identify key challenges for future research. These include, teasing apart the factors that contribute to maturation of masked speech perception including, not least, the effect of hearing status.

Walker et al. compared developmental growth rates in speech recognition for North American children with and without HL. Children with HL showed persistent deficits in masked speech recognition until the age of 11 years but their development was parallel to that of children without hearing loss. Factors that influenced growth trajectories for masked speech recognition included stronger vocabulary skills and higher hearing aid dosage. Importantly, the authors point out the need to continue to support children with hearing loss in the academic setting as they transition to secondary education. McCreery et al. investigated the effect of hearing status on masked speech perception in North American children. They found that children with HL had poorer aided speech recognition in noise and reverberation than children with typical hearing. Children with better vocabulary and working memory had better speech recognition in noise and noise plus reverberation than peers with poorer skills in these domains. In general, the better the aided audibility the better the speech recognition in noise and reverberation. McCutcheon et al. investigated the effect of musical education on masked speech perception in South African children with TD. Authors were unable to identify any effect on either speech perception or phonological short-term memory of musical education.

Some contributions in this topic use psychophysical and neuroimaging approaches to the study of possible neural correlates to challenges of listening in children with TD and HL. Moore et al. investigated dichotic listening and neural correlates of a receptive speech task in typically developing children and children with listening difficulties. There were only subtle differences between groups but while activation in some brain regions correlated with dichotic listening for the group of typically developing children this was not the case for the children with listening difficulties. In their study on children with congenital HL, Jiang et al. suggest that the changes seen in white matter microstructures could depend on poor auditory input or cortical reorganization. Cardon and Sharma examined cross-modal reorganization of the auditory cortex in children with CI using vibrotactile stimulation. They found that children with poorer speech perception in noise showed greater cross-modal reorganization, i.e., that their auditory cortices were more sensitive to vibrotactile stimulation than those with better speech perception in noise. Furthermore, greater cross-modal reorganization was seen in the cortex on the same side as their first CI indicating that this reorganization becomes more accentuated when auditory input is degraded.

To sum up, the 24 articles in this topic provide an important starting point for embracing very diverse aspects of listening difficulties in children. Key themes for further exploration are the effect of even low levels of background noise on perceived and actual listening comprehension in children, including but not limited to, children with special needs. This work should take into account developmental aspects. Also more intervention studies are called for. For example, can students' listening and language development be supported by teacher training aiming at fostering language learning i.e., vocabulary skills in the classroom? Further work is also needed on charting the neural correlates of listening difficulties in children. Last but not least, we believe that the complex relationship between the child's motivation, both intrinsic and extrinsic, and listening effort, measured both subjectively and objectively should be a key focus of future work as the development of more dynamic theoretical models of the interaction of these factors. Finally, we want to express our gratitude for all interesting contributions to this topic! They not only show the important advances we have seen in this cross-disciplinary field during the last years, but they definitely also offer a great platform for future studies.

## Author Contributions

MR initiated the topic. All four editors communicated with topic authors and reviewers during the revision process. BS was responsible for the editorial.

## Conflict of Interest

The authors declare that the research was conducted in the absence of any commercial or financial relationships that could be construed as a potential conflict of interest.

